# Long-chain PUFA and painful temporomandibular disorder in the Hispanic Community Health Study/Study of Latinos

**DOI:** 10.1017/S1368980025000102

**Published:** 2025-02-03

**Authors:** Anne E. Sanders, Jianwen Cai, Martha L. Daviglus, Olga Garcia-Bedoya, Gary D. Slade

**Affiliations:** 1 Department of Pediatric Dentistry and Dental Public Health, Adams School of Dentistry, University of North Carolina at Chapel Hill, Chapel Hill, NC 27599, USA; 2 Coordinating Center - Collaborative Studies Coordinating Center - UNC at Chapel Hill University of North Carolina at Chapel Hill, Chapel Hill, NC, USA; 3 Department of Medicine, Institute for Minority Health Research, University of Illinois at Chicago, Chicago, IL, USA

**Keywords:** Orofacial pain, Temporomandibular disorder, Hispanics/Latinos, PUFA, Dietary intake, Fish oil supplements

## Abstract

**Objective:**

*n*-6 and *n*-3 long-chain PUFA play opposing roles in inflammation, anxiety and nociception, all of which are closely associated with chronic pain. We hypothesised that diets high in *n*-6 arachidonic acid (C20:4*n*-6, AA) and low in combined *n*-3 EPA (C20:5*n*-3, EPA) and DHA (C22:6*n*-3, DHA) would be associated with higher odds of painful temporomandibular disorder (TMD).

**Design:**

We analysed baseline data from the Hispanic Community Health Study/Study of Latinos (HCHS/SOL). Two 24-h dietary recall surveys quantified intake of long-chain *n*-6 and *n*-3 PUFA along with their precursors, linoleic acid (C18:2*n*-6, LA) and alpha linolenic acid (C18:3*n*-3, ALA), respectively. *n*-3 PUFA supplementation was quantified. Interviewer-administered questions assessed TMD. Survey multiple logistic regression estimated covariate-adjusted OR and 95 % confidence limits (CL) for associations between PUFA and TMD.

**Setting:**

From 2008 to 2011, HCHS/SOL recruited 16 415 adults of Hispanic/Latino backgrounds (Cuban, Puerto Rican, Dominican, Mexican, Central/South American), through field centres located in Miami, FL; San Diego CA; Chicago, IL; and the Bronx, NY.

**Participants:**

13 870 participants with non-missing data.

**Results:**

In analysis adjusted for covariates, each sd increase in dietary intake of C20:4*n*-6, AA was associated with 12 % higher odds of TMD (OR = 1·12, CL: 1·01, 1·24). Although the dietary intake of combined long-chain C20:5*n*-3, EPA and C22:6*n*-3 DHA was not associated with TMD, each sd increase in *n*-3 dietary supplement was associated with lower odds of TMD.

**Conclusions:**

A diet rich in C20:4*n*-6, AA was associated with higher odds of painful TMD.

Temporomandibular disorder (TMD) is the most common pain complaint in the maxillofacial region, affecting 2·8 % of US men and 6·3 % of US women^(1)^, equivalent to 10 million adults. The disorder is characterised by arthralgia in the temporomandibular joint/s, myalgia in the masticatory muscles or both. On a 0–10 scale, people rate the intensity of TMD pain at 4·3 on average, the same intensity of pain as chest pain^(1)^. Painful TMD restricts chewing and mandibular movement^(2)^, and it frequently overlaps with psychological distress, hyperalgesia and other pain disorders^(3)^. US estimates of the annual costs for the diagnosis and treatment of TMD are close to $100 billion^(4)^.

In the USA, prevalence of chronic pain disorders, including TMD, has increased steeply this century^(5)^. Of note, the relative increase in TMD prevalence among adults of Hispanic origin (24 %) was approximately double the corresponding relative increases in TMD pain prevalence for non-Hispanic African American (12 %) and non-Hispanic White (13 %) adults. One explanation for the rise in chronic pain prevalence involves changes to the modern Western diet. Over the past century, advances in industrial processing made it possible to extract oils from seeds such as rapeseed, maize and soyabean – that are rich in *n*-6 PUFA. These oils, marketed as ‘vegetable oils’, were aggressively promoted as healthier substitutes for saturated fats like butter and lard. Between 1909 and 1999, the per capita consumption of *n*-6 rich soyabean oil rose more than 1000-fold^(6)^. At the same time, consumption of *n*-3 PUFA, obtained from fatty fish, declined. This shift resulted in a dramatic imbalance in the dietary intake of *n*-6 and *n*-3 PUFA, such that the *n*-6/*n*-3 PUFA ratio in the average diet increased from a balanced 4:1 to a highly skewed 20:1^(7)^. The high consumption of seed oils such as maize, sunflower and soyabean in the modern Western diet leads to elevated intake of C18:2*n*-6, linoleic acid (LA), the precursor for C20:4*n*-6, arachidonic acid (AA). This results in increased circulating levels of C20:4*n*-6, AA, which can impact various physiological processes, including inflammation and pain signalling.

The synthesis of *n*-6 and *n*-3 PUFA begins with the two essential fatty acids, LA (C18:2*n*-6) and alpha linolenic acid (ALA) (C18:3*n*-3), both of which must be obtained from the diet as the human body cannot synthesise them. C18:2*n*-6, LA is converted to C20:4*n*-6, AA through the action of delta-6 desaturase (D6D) and delta-5 desaturase (D5D), whereas C18:3*n*-3, ALA acid is converted to C20:5*n*-3, EPA and C22:6*n*-3, DHA primarily via D6D and elongase-2 (ELOVL2). PUFA synthesis primarily occurs in the liver but can also take place in adipose tissue and the brain. Diet plays a critical role in PUFA synthesis, as high intake of the essentially fatty acids promotes it, while saturated fats may inhibit the process. Additionally, vitamins and minerals support enzyme function essential for synthesis, whereas oxidative stress can impair desaturase activity. Hormonal factors, particularly insulin and thyroid hormones, also modulate enzyme activity, further influencing PUFA synthesis.

*n*-6 and *n*-3 PUFA play opposing roles in physiological processes involving inflammation, nociception and anxiety. In general, *n*-6 derivatives amplify these effects^(8,9)^, whereas *n*-3 derivatives inhibit them^(10–12)^. An imbalanced PUFA ratio favouring *n*-6 is implicated in the pathophysiology of several inflammatory diseases including CVD^(13)^, neurodegenerative disease such as Alzheimer’s disease^(14)^, psychiatric disorders such as major depressive disorder^(15)^, and pain disorders including migraine^(16)^, chronic daily headache^(12)^, rheumatoid arthritis^(17)^ and osteoarthritis^(18)^. A recent clinical study showed that higher circulating levels of *n*-3 EPA (C20:5*n*-3, EPA) and DHA (C22:6*n*-3, DHA) were associated with lower probability of painful TMD^(19)^ and lower pain thresholds^(20)^, whereas higher circulating levels of AA (C20:4*n*-6) and a higher *n*-6/*n*-3 PUFA ratio were associated with greater probability of painful TMD^(20)^. It remains unclear whether similar associations between PUFA and TMD exist when PUFA are assessed through dietary intake measures rather than in blood. Furthermore, this association has not been studied in the diverse Hispanic population, whose diets often differ from the standard American diet.

We sought to better understand the relationship between dietary intake of PUFA and TMD in adults with Hispanic/Latino background. The two hypotheses are that diets rich in *n*-6 PUFA would be associated with higher odds of TMD and that diets rich in *n*-3 PUFA would be associated with lower odds of TMD.

## Methods

### Research design and study population

This study is a cross-sectional analysis of baseline data from the Hispanic Community Health Study/Study of Latinos (HCHS/SOL). The HCHS/SOL is a community-based, multicentre prospective cohort study of 16 415 self-identified Hispanic/Latino adults aged 18–74 years at baseline in 2008–2011. A multistage probability sampling design recruited participants from randomly sampled census block areas within four US areas located in the Bronx, New York; San Diego, California; Miami, Florida; and Chicago, Illinois^(21,22)^. Participants included adults with Cuban, Dominican, Mexican, Puerto Rican, and Central and South American backgrounds. Enrolled participants completed an in-person interview with trained assessors in their preferred language (English or Spanish) in which they reported sociodemographic characteristics of age, sex, nativity status and Hispanic/Latino heritage.

### Temporomandibular disorder pain

TMD was assessed using the interviewer-administered Oral Health Questionnaire in which participants were asked whether they had experienced pain in their face, and whether they had experienced pain in their jaw joint, within the past 12 months. Response options for both questions were yes or no. Other questions were asked to eliminate sources of other orofacial pain, such as tooth ache. To be classified as a TMD case, participants had to report having had pain in both their face and in their jaw joint.

The requirement of pain in both face and jaw joint is a stringent case definition consistent with the predominant findings that most people with chronic TMD have both myalgia and arthralgia when evaluated clinically^(23)^. People without pain in their face and jaw are defined as TMD non-cases.

### Dietary PUFA and total energy intake

Dietary intake was assessed in two 24-h dietary recalls^(24)^. The first recall was administered at the baseline HCHS/SOL clinic visit by in-person interview and the second by telephone approximately 30 d later. Each interview was conducted either in Spanish language (80 % of participants) or in English language (20 % of participants), according to each participant’s preference. In each interview, participants estimated portion sizes with the use of food models (in-person) or a food-amount booklet (for telephone interviews). Data on foods and nutrients were determined using the multiple-pass methods of Nutrition Data System for Research software (version 11) from the Nutrition Coordinating Center at the University of Minnesota. Dietary intake was determined by averaging the two 24-h dietary recalls. Estimated usual intake distributions were modelled by using the NCI method^(25)^ with SAS software macros (version 9.3; SAS Institute) developed at the NCI. This method allowed for the estimation of within- and between-person variance components and correction for the high intraindividual variation intrinsic to 24-h recalls.

The major long-chain *n*-6PUFA is AA, and the major long-chain *n*-3 PUFA are EPA and DHA. Our rationale for combining EPA and DHA in this analysis is because: marine fatty fish is the food source for both; and because EPA and DHA are combined in recommendations of dietary intake levels^(26,27)^. Total energy intake (kcal) was calculated from both dietary recalls, and values that fell below the 1st or above the 99th percentile by sex were excluded (*n* 617, 1·9 %)^(28)^. Dietary recalls were also excluded that interviewers considered unreliable because of missing or unrealistic values.

### *n*-3 fatty acid (fish oil) dietary supplement use

Dietary supplements were assessed in the two 24-h dietary recalls and with two additional methods: an in-person Dietary and Supplement Recall Interview to which participants brought all the dietary supplements and home remedies they had taken in the past four weeks and a medication inventory. The Dietary Supplements assessment module of the Nutrition Data System for Research classified these supplements. In this analysis, we used a continuous variable of long-chain *n*-3 fatty acid supplement measured in milligrams (mg) per d.

### Covariates

Selection of covariates was guided by evidence of their associations with PUFA and painful TMD. Inclusion of these covariates in multivariable analysis avoids confounding bias, allowing us to better isolate their influence on TMD.

### Alternate Healthy Eating Index

The Alternate Healthy Eating Index-2010 (AHEI-2010) is a measure of dietary quality according to the 2010 Dietary Guidelines for Americans^(29)^ that assigns ratings to foods and nutrients predictive of chronic disease. The index is based on eleven components: six components for which highest intakes are ideal, one component for which moderate intake is ideal and four components for which lowest intake is ideal. Each component is scored accordingly, and component scores are summed to obtain a total AHEI-2010 score, which ranges from 0 to 110, with a higher score representing a healthier diet. Adjusting for the AHEI score helps clarify the relationship between dietary acids and TMD by controlling for potential confounding of dietary quality, leading to more valid and reliable conclusions.

### BMI, smoking status and physical activity

Chronic pain is associated with higher BMI, cigarette smoking and a sedentary lifestyle. Anthropometric measurements were performed during the examination by trained and certified staff following a standard protocol. Height was measured to the nearest centimetre and body weight to the nearest 0·1 kg to determine BMI as height in kilograms divided by the square of measured height in metres. Cigarette smoking status was measured by self-report and categorised into three categories: current smoker, former smoker and never smoked. The Global Physical Activity Questionnaire (GPAQ) was interviewer-administered to record minutes of physical activity in a typical week. This questionnaire is both valid and reliable in capturing self-reported physical activity^(30)^. In this analysis, total physical activity was computed as time spent in moderate and vigorous physical activity converted into metabolic equivalent task units (MET) and expressed as MET-min/week. A MET is computed as the ratio of working metabolic rate relative to RMR with metabolic rate defined as the rate of energy expended per unit of time. The recommended expenditure of MET min per week for good health is between 450 and 750. The American Heart Association recommends at least 150 min of moderate-intensity aerobic exercise each week, equivalent to 500 MET minutes per week^(31)^.

### Symptoms of depression

Meta-analyses of randomised controlled trials conclude that *n*-3 PUFA are therapeutic for psychological distress^(32)^, and psychological distress is one of the strongest predictors of first-onset TMD^(33)^. The ten-item Center for Epidemiologic Studies Depression (CESD-10) scale is the short form derived by Andresen et. al.^(34)^, from the full twenty-item CESD scale. It is designed to screen for depressive symptoms. Scores have a potential range of 0 to 30, with scores of 10 or more classifying people as positive for depressive symptoms^(35)^. The CESD-10 scale shows good predictive accuracy of the full-length twenty-item version of the scale (κ = 0·97) and good validity and reliability^(34)^ in detecting depressive symptoms in both clinical and non-clinical populations. In the HCHS/SOL study population, the psychometric properties of the CES-D 10 total score showed acceptable internal consistencies (Cronbach *α*’s = 0·80–0·86) and test–retest reliability (r’s = 0·41–0·70) across the full sample, language group and ethnic background group^(36)^.

### Statistical analyses

Analytic methods for a complex sample survey were used to account for the clustered sampling and the use of stratification in sample selection as well as to incorporate sampling weights to account for the disproportionate selection of the sample^(21)^. Sampling weights were calibrated to the 2010 US Census according to age, sex and Hispanic/Latino heritage. Of the 16, 415 participants, the current analysis was restricted to dentate adults who completed the Oral Health Questionnaire (*n* 15 140) and had reliable 24-h dietary recalls and no missing values on covariates. Hence, these analyses included data from 13 870 participants. Subpopulation analyses provided variance estimates that were unbiased by the excluded observations. All analyses were performed using Stata/se release 14.2 (Stata Corp.).

For descriptive analyses, we first examined associations between study participant characteristics and their daily dietary intake of PUFA. We applied established cut points for BMI and the CESD-10 and constructed tertiles of diet quality score, energy intake and MET minutes per week. In multivariable analyses, these independent variables were modelled as continuous scores. We next compared the dietary intake of PUFA and other study characteristics between adults with and without TMD, highlighting similarities and differences.

For the primary analysis of associations, survey logistic regression analysis estimated OR and 95 % confidence limits (95 % CL) for the association between PUFA and TMD. Multivariable models adjusted for potential confounding of factors likely to influence PUFA intake and TMD. Specifically, regression models adjusted for field centre, age in years, sex, heritage, educational attainment, *n*-3 dietary supplement intake, Alternative Healthy Eating Index 2010 score (AHEI-10), total energy (kilojoules (kJ)/d), smoking status, BMI, total physical activity (MET-min/week) and psychological distress (CESD-10 depressive symptom score). In multivariable models, continuous variables were standardised as z-scores to enable their normative interpretations as the change in odds of TMD per sd change in the independent variable.

Exploratory analysis investigated potential effect modification by evaluating the association of C20:4*n*-6, AA and TMD across different levels of C20:5*n*-3, EPA plus C22:6*n*-3, DHA. This was achieved by adding an interaction term of C20:4*n*-6, AA and C20:5*n*-3, EPA plus C22:6*n*-3, DHA to the primary analytic model described above. The two-way contour command in Stata was used to visualise the effect modification of the *n*-3 PUFA on the relationship between *n*-6 on TMD. This produced a contour plot in which the continuous measures of C20:5*n*-3, EPA plus C22:6*n*-3, DHA and C20:4*n*-6, AA were presented on the Y and X axes, respectively, and the predicted probability of TMD was depicted using colour shading. The precursors to long-chain PUFA were not included in this analysis. Estimates adjusted for field centre, sex, background, educational attainment and baseline values of age in years, *n*-3 dietary supplement intake, AHEI-10, total energy (kilojoules (kJ)/d), smoking status, BMI, total physical activity (MET-min/d) and the total summary score on the ten-item CESD-10 scale.

## Results

The average daily intake of C18:2*n*-6, LA (Table 1) was more than 160 % of combined C20:5*n*-3, EPA plus C22:6*n*-3, DHA. Both C18:2*n*-6, LA and C20:4*n*-6, AA were positively associated with energy intake, physical activity and cigarette smoking but inversely associated with age and depressive symptoms. *n*-3 intake was positively associated with educational attainment, diet quality, energy intake and physical activity but inversely associated with depressive symptoms. Of the 6·4 % of adults using *n*-3 supplements, daily intake was positively associated with age and educational attainment and was higher in non-current smokers than in smokers. That supplement use was also higher in people with more depressive symptoms suggests that these people may seek out *n*-3 supplements as a complementary treatment or preventive measure for psychological distress.

**Table 1.** Daily dietary intake (mg) of PUFA by sociodemographic characteristics, HCHS/SOL visit 1 (2008–2011), unweighted *n* 13 870

Characteristic	%	C18:2*n*-6, LA		C20:4*n*-6, AA		C18:3*n*-3, ALA		C20:5*n*-3, EPA C22:6*n*-3, DHA		*n*-3 PUFA in supplements	
Total	100·0	14 595·1	*P*	155·5	*P*	155·4	*P*	87·7	*P*	28·7	*P*
Sex											
Women (ref)	51·9	12 158·8	< 0·001	124·1	< 0·001	131·0	< 0·001	73·0	< 0·001	31·8	0·099
Men	48·1	17 220·0		189·3		181·6		103·6		25·4	
Age group (years)											
18–34 (ref)	40·2	15 982·6	< 0·001	162·7	< 0·001	166·7	< 0·001	86·5	0·005	8·2	< 0·001
35–44	21·9	15 383·4		161·6		163·9		89·8		21·3	
45–54	18·9	13 901·0		158·4		147·0		89·7		42·6	
≥ 55	19·0	11 442·4		130·2		129·9		86·0		66·9	
Heritage											
Dominican	9·0	13 093·5	< 0·001	158·0	ns	137·4	< 0·001	103·5	< 0·001	31·1	0·011
Central American	7·4	14 501·9		158·9		156·5		83·5		38·6	
Cuban	20·0	16 099·1		157·0		182·2		79·6		44·6	
Mexican	39·8	14 406·1		151·2		148·5		87·6		19·8	
Puerto Rican	14·5	14 142·5		153·8		144·7		79·6		16·5	
South American (ref)	5·0	13 666·9		157·6		156·3		112·4		34·6	
> 1 Heritage/Other	4·3	15 255·0		180·0		164·4		99·8		49·9	
Educational attainment											
< High school diploma	31·0	13 399·3	< 0·001	150·0	ns	138·8	< 0·001	85·3	< 0·001	27·9	0·029
High school diploma	29·1	15 485·6		159·7		163·9		86·7		22·6	
> High school diploma (ref)	39·9	14 876·1		156·6		162·0		90·4		33·9	
AHEI-10 diet quality											
Lowest tertile	33·4	14 884·8	0·031	155·4	ns	162·5	0·002	77·3	< 0·001	21·2	< 0·001
Intermediate tertile	33·3	14 847·2		156·2		155·9		88·2		26·3	
Highest tertile (ref)	33·3	14 053·2		154·8		147·8		97·7		38·8	
Energy intake (kcal)											
Lowest tertile (ref)	33·3	6963·3	< 0·001	95·3	< 0·001	74·8	< 0·001	78·3	< 0·001	32·0	0·037
Intermediate tertile	33·3	13 277·0		143·9		141·9		87·1		31·1	
Highest tertile	33·3	23 546·0		227·2		249·5		97·9		23·2	
BMI (kg/m^2^)											
Underweight/normal (< 25) (ref)	23·2	15 632·2	< 0·001	156·6	ns	165·4	0·007	85·9	< 0·001	15·5	< 0·001
Overweight (25·0 to < 30)	37·4	14 191·0		156·1		152·4		90·1		32·2	
Obese (≥ 30)	39·4	14 368·6		154·2		152·3		86·5		33·2	
Cigarette smoking status											
Never smoked (ref)	62·4	13 668·0	< 0·001	146·6	< 0·001	145·7	< 0·001	85·6	< 0·001	30·4	< 0·001
Former smoker	16·9	14 789·9		165·3		157·1		93·0		37·8	
Current smoker	20·7	17 228·6		174·2		183·2		89·9		16·3	
Physical activity (MET min/week)											
Lowest tertile (ref)	34·0	13 494·4	< 0·001	147·3	< 0·001	143·8	< 0·001	82·3	< 0·001	33·1	< 0·001
Intermediate tertile	32·7	13 848·5		148·8		148·4		87·0		33·2	
Highest tertile	33·3	16 451·0		170·3		174·1		94·0		19·9	
CESD-10 depressive symptoms											
Not depressed (0–< 10) (ref)	73·6	14 830·3	0·038	158·3	0·010	158·2	0·034	89·4	< 0·001	27·0	ns
Depressed (10–30)	26·4	13 938·9		147·5		147·4		83·0		33·7	

HCHS/SOL, Hispanic Community Health Study/Study of Latinos; LA, linoleic acid; AA, arachidonic acid; ALA, alpha linolenic acid; AHEI-10, Alternate Healthy Eating Index-2010; MET, metabolic equivalent; CESD, Center for Epidemiologic Studies Depression.

Prevalence of TMD in this study population was 5·1 % (Table 2), equivalent to TMD prevalence in the US population overall. In these unadjusted associations, dietary intake of C18:2*n*-6, LA and C20:4*n*-6, AA were not associated with TMD, whereas intake of long-chain *n*-3 PUFA and *n*-3 supplement use were lower in TMD cases (16·9 mg) than in non-cases (29·4 mg). As seen in other studies of painful TMD, more women than men had the condition, as did adults with less educational attainment and people with higher scores for depressive symptoms.

**Table 2.** Descriptive characteristics by TMD status, HCHS/SOL 2008–2011, unweighted *n* 13 870

Characteristic	TMD cases *n* 873		TMD non-cases *n* 12 997		*P*-value
Total; % (se)	5·1	0·3	94·9	0·3	
Linoleic acid (C18:2*n*-6, LA), mg; mean (se)	13996·1	566·4	14627·1	186·2	0·291
Alpha-linolenic acid (C18:3*n*-3, ALA), mg; mean (se)	139·9	6·3	156·2	2·3	0·013
Arachidonic acid (C20:4*n*-6, AA), mg; mean (se)	156·1	7·3	155·4	2·0	0·926
C20:5*n*-3, EPA plus C22:6*n*-3, DHA, mg; mean (se)^*^	81·7	1·7	88·1	0·6	< 0·001
*n*-3 supplement, mg; mean (se)	16·9	4·4	29·4	2·1	0·008
Sex; % (se)					
Women	6·6	0·4	93·4	0·4	< 0·001
Men	3·4	0·3	96·6	0·3	
Age, years; mean (se)	42·8	0·7	40·1	0·3	< 0·001
Heritage; % (se)					
Dominican	4·5	0·7	95·5	0·7	0·033
Central American	5·8	0·6	94·2	0·6	
Cuban	4·2	0·7	95·8	0·7	
Mexican	5·0	0·5	95·0	0·5	
Puerto Rican	6·9	0·7	93·1	0·7	
South American	3·8	0·6	96·2	0·6	
> 1 Heritage/Other	5·1	1·1	94·9	1·1	
Educational attainment; % (se)					
Less than high school diploma	5·9	0·4	94·1	0·4	0·043
High school diploma	4·5	0·4	95·5	0·4	
More than high school diploma	4·9	0·4	95·1	0·4	
AHEI-10 score, mean (se)^†^	47·5	0·4	47·5	0·2	0·983
Energy intake, kcal; mean (se)	8199·8	187·5	8811·9	62·3	0·001
BMI, kg/m2; mean (se)	30·0	0·3	29·3	0·1	0·035
Smoking status; % (se)					
Never smoked	4·8	0·3	95·2	0·3	0·058
Former smoker	4·8	0·5	95·2	0·5	
Current smoker	5·1	0·3	94·9	0·3	
Physical activity, MET min/week; mean (se)^‡^	656·3	49·0	718·3	16·9	0·229
CESD-10 depressive symptoms; mean (se)^§^	10·2	0·3	6·7	0·9	< 0·001

TMD, temporomandibular disorder; HCHS/SOL, Hispanic Community Health Study/Study of Latinos; LA, linoleic acid; ALA, alpha linolenic acid; AA, arachidonic acid; AHEI-10, Alternate Healthy Eating Index-2010; MET, metabolic equivalent; CESD, Center for Epidemiologic Studies Depression.

* Combined EPA (20:5*n*-3, EPA) and DHA (C22:6*n*-3, DHA).

† The AHEI-10 is a score comprised of eleven food and nutrient components, ranging from 0 to 110 (unhealthiest to healthiest diet quality).

‡ A MET is the ratio of the working metabolic rate relative to the RMR.

§ The CESD-10 item scale.

In the multivariable logistic regression analysis that examined associations of PUFA with TMD adjusted for sociodemographic characteristics (Table 3, model 1), each sd increment in C20:4*n*-6, AA intake was associated with 11 % higher odds of TMD (OR = 1·11, 95 % CL: 1·01, 1·21). After further adjustment for diet quality, energy intake, BMI, smoking status, physical activity and depressive symptoms (model 2), the association remained unchanged (aOR = 1·12, 95 % CL: 1·01, 1·24, *P* = 0·025). In this fully adjusted model, each sd increment in *n*-3 supplement intake was associated with 14 % lower odds of TMD (aOR = 0·86, 95 % CL: 0·78, 0·96, *P* = 0·006). Central Americans and Puerto Ricans retained higher odds of TMD than South Americans.

**Table 3.** Multivariable-adjusted logistic regression odds ratios and 95 % confidence limits (CL)) of associations between *n*-3 and *n*-6 PUFA dietary intake and TMD, HCHS/SOL 2008–2011, *n* 13 870

	Model 1			Model 2		
	OR	95 % CI	*P*	OR	95 % CI	*P*
Linoleic acid (C18:2*n*-6, LA)	1·14	0·97, 1·35	0·106	1·16	0·95, 1·41	0·142
Arachidonic acid (C20:4*n*-6, AA)	1·11	1·01, 1·21	0·029	1·12	1·01, 1·24	0·025
Alpha-linolenic acid (C18:3*n*-3, ALA)	0·85	0·70, 1·02	0·087	0·86	0·71, 1·04	0·126
C20:5*n*-3, EPA plus C22:6*n*-3, DHA^*^	0·97	0·85, 1·10	0·620	0·97	0·84, 1·12	0·701
*n*-3 dietary supplement	0·87	0·78, 0·96	0·008	0·86	0·78, 0·96	0·006
Sex						
Women	2·04	1·61, 2·58	< 0·001	1·71	1·32, 2·21	< 0·001
Men	Ref			Ref		
Age	1·01	1·01, 1·02	< 0·001	1·01	1·00, 1·02	0·003
Heritage						
Dominican	1·26	0·74, 2·13	0·392	1·26	0·73, 2·16	0·411
Central American	1·61	1·05, 2·48	0·030	1·65	1·05, 2·58	0·030
Cuban	1·26	0·75, 2·11	0·379	1·14	0·67, 1·94	0·618
Mexican	1·12	0·70, 1·79	0·646	1·23	0·72, 2·08	0·446
Puerto Rican	1·72	1·09, 2·73	0·021	1·39	0·86, 2·24	0·180
South American	Ref			Ref		
> 1 Heritage/Other	1·51	0·85, 2·71	0·161	1·43	0·79, 2·60	0·241
Educational attainment						
Less than high school diploma	1·11	0·88, 1·39	0·370	0·94	0·75, 1·19	0·624
High school diploma	0·95	0·75, 1·22	0·707	0·87	0·68, 1·11	0·253
More than high school diploma	Ref			Ref		
AHEI-10 diet quality^†^				0·97	0·82, 1·15	0·736
Energy intake				0·92	0·79, 1·09	0·350
BMI				1·02	0·92, 1·12	0·750
Smoking status						
Never smoked				Ref		
Former smoker				1·00	0·77, 1·29	0·974
Current smoker				1·25	0·98, 1·60	0·079
MET min/week^‡^				1·04	0·94, 1·15	0·427
CESD-10 depressive symptoms^§^				1·56	1·43, 1·70	< 0·001
Intercept	0·01	0·01, 0·02	< 0·001	0·01	0·01, 0·03	< 0·001

TMD, temporomandibular disorder; HCHS/SOL, Hispanic Community Health Study/Study of Latinos; LA, linoleic acid; AA, arachidonic acid; ALA, alpha linolenic acid; AHEI-10, Alternate Healthy Eating Index-2010; MET, metabolic equivalent; CESD, Center for Epidemiologic Studies Depression.

Both models adjust for field centre (not reported). Model 1 additionally adjusts for sex, age, heritage and educational attainment. Model 2 additionally adjusted for diet quality, energy intake, BMI, smoking status, physical activity and depressive symptoms. Continuous covariates of fatty acids, *n*-3 supplementation, AHEI-10 diet quality, energy intake, BMI, physical activity and depressive symptoms are standardised to z-scores.

The abbreviation Ref refers to the referent category for the categorical variable.

* Combined EPA (20:5*n*-3) and DHA (C22:6*n*-3).

† The AHEI-10 is a score comprised of eleven food and nutrient components, ranging from 0 to 110 (unhealthiest to healthiest diet quality).

‡ A MET is the ratio of the working metabolic rate relative to the RMR.

§ The CESD-10 item scale.

**Figure 1.**
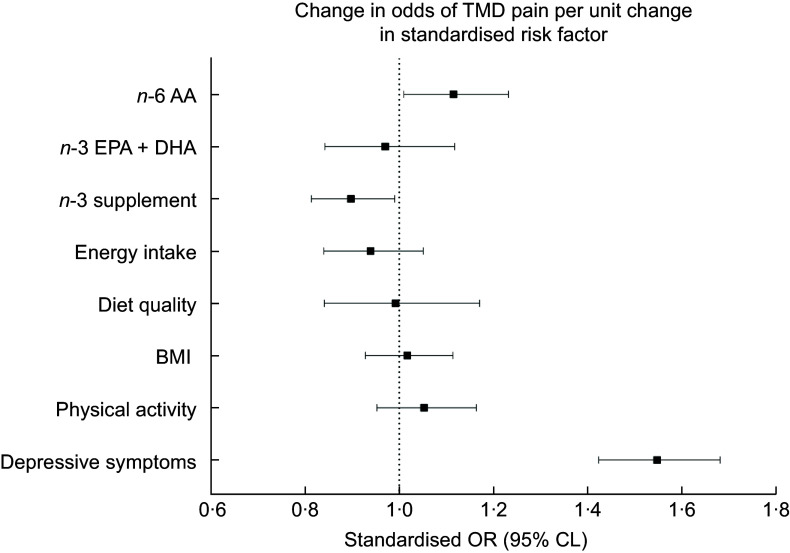
Estimates are the covariate-adjusted change in odds of TMD (95 % CL) per sd increment in key independent variables, taken from a multiple logistic regression model in which the dependent variable is TMD. Independent variables were z-standardised to a mean of 0 and a sd of 1, to compare their relative associations with TMD. The model additionally adjusts for field centre, sex, age in years, heritage, educational attainment and smoking status (unplotted). Diet quality was assessed with Alternative Healthy Eating Index 2010 score (AHEI-10), energy intake (kilojoules (kJ)/d) was assessed in two 24-h dietary recalls, total physical activity (MET-min/week) was assessed with the Global Physical Activity Questionnaire (GPAQ) and depressive symptoms was assessed with the 10-item Center for Epidemiologic Studies Depression (CESD-10) scale. Estimates that cross the null value of 1·0 are not statistically significantly associated with TMD. TMD, temporomandibular disorder; CL, confidence limit; MET, metabolic equivalent.

A forest plot (Figure 1) of estimates from model 3 enables visual comparison of the direction, effect size and precision of the key standardised risk/protective factors for TMD. The most pronounced of these was the positive association between depressive symptoms and TMD.

**Figure 2.**
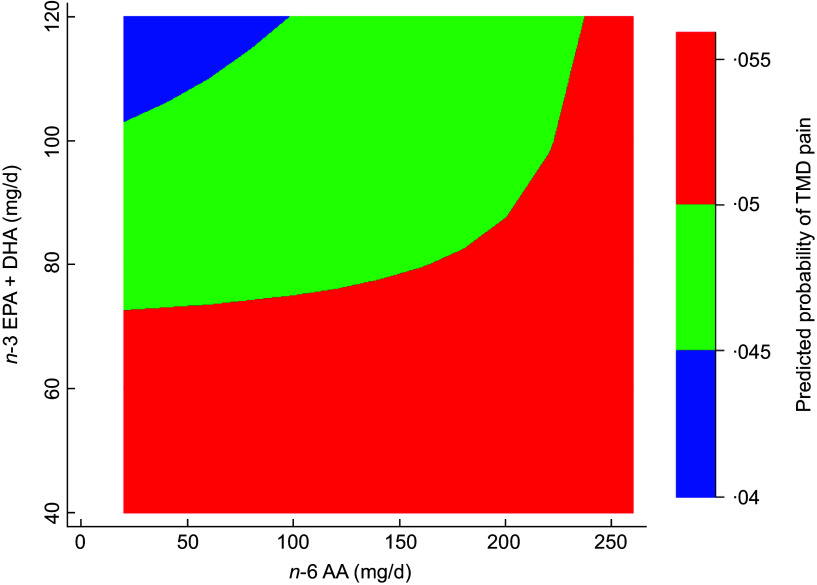
A contour plot showing the effect modification (*P* < 0·001) between long-chain *n*-6 C20:4*n*-6, AA and *n*-3 C20:5*n*-3, EPA plus C22:6*n*-3, DHA on predicted probability of TMD. The colour scheme indicates discrete levels of probabilities of TMD. Of note, all adults with low intake of EPA + DHA had high probability of TMD, irrespective of their AA intake (red area). Estimates are from a multiple logistic regression model that adjusted for field centre, sex, heritage, educational background and baseline values of age in years, *n*-3 dietary supplement intake, Alternative Healthy Eating Index 2010 score (AHEI-10), total energy (kilojoules (kJ)/d), smoking status, BMI, total physical activity (MET-min/d) and the total summary score on the 10-item Center for Epidemiologic Studies Depression (CESD-10) scale. AA, arachidonic acid; TMD, temporomandibular disorder.

The contour plot from the exploratory analysis (Figure 2) depicts effect modification between the two classes of long-chain PUFA in association with TMD. Of note, all adults with low EPA + DHA intake had high probability of TMD, irrespective of their AA intake (red area).

## Discussion

In this large, community-based study of Hispanic/Latino men and women, diets rich in long-chain C20:4*n*-6, AA were associated with higher odds of TMD, independent of *n*-3 dietary intake and supplementation. Specifically, each sd increment in C20:4*n*-6, AA intake was associated with 12 % higher in covariate-adjusted odds of TMD. Furthermore, each sd increment in *n*-3 supplement intake was associated with 14 % lower covariate-adjusted odds of TMD.

In this study population, C20:4*n*-6, AA dietary intake was associated with greater odds of TMD. C20:4*n*-6, AA is a precursor to pro-inflammatory molecules such as prostaglandins and leukotrienes that play a role in pain signalling pathways. Boyd et al.^(8)^ demonstrated that mice administered a diet resembling the modern Western diet rich in *n*-6 PUFA developed persistent nociceptive hypersensitivities and peripheral neuropathy after 8 weeks. Reversing the diet with high levels of *n*-3 PUFA attenuated the nociceptive behaviours and neurophysiologic abnormalities induced by the high *n*-6 intake. Our own clinical observation study used pressure algometry to test pressure pain thresholds. In study participants, higher circulating levels of *n*-6 LA and AA were associated with lower pressure pain thresholds (meaning higher pain sensitivity) at multiple anatomical sites^(20)^.

A high intake of C18:2*n*-6, LA can reduce the availability of long-chain C20:5*n*-3, EPA and C22:6*n*-3, DHA due to competition for the same metabolic enzymes. Both *n*-6 and *n*-3 fatty acids share common enzymes, particularly D6D and D5D, which are responsible for converting their respective precursor fatty acids (C18:2*n*-6, LA to C20:4*n*-6, AA, and C18:3*n*-3, ALA to C20:5*n*-3, EPA and C22:6*n*-3, DHA). When dietary intake of C18:2*n*-6, LA is high, it can upregulate the activity of these enzymes, diverting metabolic resources towards the *n*-6 pathway and thus limiting the conversion of C18:3*n*-3, ALA to C20:5*n*-3, EPA and C22:6*n*-3, DHA. This results in a lower availability of *n*-3 PUFA, which are essential for various physiological functions, including anti-inflammatory processes.

Counter to our hypothesis, we did not find that a higher intake of long-chain *n*-3 PUFA was inversely associated with TMD. One possible explanation is that the average dietary intake of long-chain *n*-3 PUFA in this population may be too low to exert a measurable effect. The combined C20:5*n*-3, EPA plus C22:6*n*-3, DHA intake was 88 mg/d on average. In comparison, in the 2017–2018 US National Health and Nutrition Examination Survey (NHANES), adults’ mean daily intake of C20:5*n*-3, EPA plus C22:6*n*-3, DHA was 111 mg^(37)^. Yet this intake is also low by international standards. For example, in a systematic review of dietary fat consumption that used data from 1 630 069 adult participants in 266 surveys conducted in 187 countries, 83 % of which used nationally representative samples. It reported a global mean intake of C20:5*n*-3, EPA plus C22:6*n*-3, DHA of 163 mg/d^(38)^ nearly double the daily intake of the HCHS/SOL cohort. The authors noted that in 100 nations, predominantly in South America, sub-Saharan Africa and Asian mainland regions, C20:5*n*-3, EPA plus C22:6*n*-3, DHA intake levels were rated extremely low (< 100 mg/d), equivalent to intake observed in this HCHS/SOL study population.

In the HCHS/SOL study population, 6·4 % of HCHS/SOL participants took an *n*-3 PUFA (fish oil) supplement at baseline. Supplement usage was inversely associated with TMD, even after adjusting for demographic characteristics, diet quality, energy intake, physical activity, smoking status and depressive symptoms. Wide variability exists in the dose of EPA + DHA in commercially available *n*-3 supplements, but an assessment of 282 unique labels found that the median (IQR) dose was 340 (135–647) mg/d for EPA, 270 (140–500) mg/d for DHA and 600 (300–1100) mg/d for combination EPA + DHA^(39)^, well above the level obtained in modern diets. The potential pain-reducing benefits of *n*-3 PUFA may be attributable to potent lipid derivatives of long-chain C20:5*n*-3, EPA and C22:6*n*-3, DHA. In particular, specialised proresolving mediators, such as resolvins, protectins and maresins, can lower the activity of the transcription factor NF-kB, which plays a key role in the inflammatory response. Modulation of inflammation is one likely mechanism by which these lipid mediators may contribute to the alleviation of TMD^(40)^.

According to the 2017–2018 NHANES, *n*-3 supplements are the third most common dietary supplement in the US adult population, ranked below multivitamin-minerals and vitamin D. *n*-3 supplement use increases with age, being taken by 5·4 % of adults younger than 40, 12·5 % aged 40–59 years and 21·8 % aged 60 years and older^(41)^. In a meta-analysis of the analgesic effects of *n*-3 PUFA supplements on chronic inflammatory pain, Goldberg & Katz^(42)^ synthesised evidence from seventeen randomised controlled trials studying adults with rheumatoid arthritis or joint pain secondary to inflammatory bowel disease and dysmenorrhea. They found that *n*-3 PUFA supplementation significantly reduced joint pain intensity, number of painful and/or tender joints, and NSAID consumption.

A recent network meta-analysis of forty randomised controlled trials of pharmacological options for migraine prophylaxis revealed that a high dose of long-chain *n*-3 supplementation had the highest efficacy and highest acceptability of all studied treatments^(43)^. Not only is this finding novel and clinically important, it has implications for TMD, as both conditions involve heightened sensitivity to pain, and abnormalities in pain processing, and share muscle tension and symptom overlap.

These dietary intake findings from the HCHS/SOL study population build on our OPPERA prospective cohort study findings in which circulating levels of PUFA in erythrocytes were quantified by liquid chromatography-tandem MS and TMD was clinically verified according to established diagnostic criteria^(44)^. OPPERA showed that a higher *n*-6/*n*-3 PUFA ratio was strongly associated with TMD, as well as the intensity of pain from TMD and overlapping chronic pain conditions^(45)^ and higher levels of psychological distress^(46)^. Furthermore, OPPERA showed that psychological distress predicted future onset of TMD and was associated with TMD persistence^(47)^.

To date, most clinical studies of dietary intake of PUFA and pain have relied on observational study evidence. One exception was a randomised controlled trial that tested the analgesic effect of a dietary intervention in 182 adults with chronic migraine. Adults were randomised to 1 of 2 dietary regimens or a control diet for 16 weeks^(16)^. One regimen increased combined C20:5*n*-3, EPA and C22:6*n*-3, DHA intakes to 1·5 g/d and maintained *n*-6 LA at 7 % of energy intake, equivalent to the average US intake. The other regimen increased EPA and DHA intakes to 1·5 g/d and decreased *n*-6 LA to < 1·8 % of energy intake. The control diet held all three PUFA intakes at average US levels. Compared with the control diet, both interventions decreased frequency and severity of headaches and increased levels of 17-HDHA, which is an antinociceptive lipid derivative of DHA.

The USA and Canada presently have no specific nutrient recommendations for C20:5*n*-3, EPA and C22:6*n*-3, DHA. This is unexpected given the strength of current evidence for these long-chain fatty acids in reducing CVD and metabolic disease risk^(48)^ and severity of depression^(32)^. However, the 2015–2020 Dietary Guidelines for Americans^(49)^ and the Scientific Report of the 2015 US Dietary Guidelines Advisory Committee^(50)^ recommend at least two fish servings of fish, preferably oily fish, per week.

This study has several limitations. HCHS/SOL’s hybrid sampling design of probability sampling from within pre-selected cities is more rigorous than simple random sampling, but generalisability to the broader Hispanic/Latino population is still limited. The cross-sectional study design cannot resolve temporal ambiguity or permit causal inference. For example, participants with TMD myalgia or arthralgia may take *n*-3 supplements to relieve pain, while TMD-free participants may take *n*-3 supplements to reduce future risk of chronic pain. We can only assess associations between supplement use and TMD, without knowing whether supplements are therapeutic or prophylactic. Self-assessed TMD is prone lacks the precision of clinical examination following diagnostic criteria for TMD and may be influenced by language barriers and cultural norms about pain tolerance. On the other hand, the HCHS/SOL’s TMD questions asked are like those used in the NHIS and have good face, criterion and discriminant validity. Measurement error is also inherent in assessing dietary intake by recall. This method overcomes several weaknesses inherent in dietary recall by accounting for day-to-day variability and food eaten infrequently.

Despite these limitations, the study has major strengths. It is the first study of TMD in a large Hispanic/Latino population with sufficient representation of heritage groups to examine differences between groups, which is not possible in national surveys like the NHANES and the NHI. It is also the first epidemiologic study reporting associations between dietary intake of *n*-6 and *n*-3 PUFA and TMD. When taken together with earlier clinical findings^(20)^, these observational study findings support the need for a randomised, placebo-controlled trial to test the analgesic efficacy of a dietary supplement of *n*-3 PUFA in adults with painful TMD.
